# Pharmacogenomics of Drugs Used in β-Thalassemia and Sickle-Cell Disease: From Basic Research to Clinical Applications

**DOI:** 10.3390/ijms25084263

**Published:** 2024-04-12

**Authors:** Roberto Gambari, Aliyu Dahiru Waziri, Hemali Goonasekera, Emmanuel Peprah

**Affiliations:** 1 Center “Chiara Gemmo and Elio Zago” for the Research on Thalassemia, Department of Life Sciences and Biotechnology, Ferrara University, 40124 Ferrara, Italy; 2 Department of Hematology and Blood Transfusion, Ahmadu Bello University Teaching Hospital Zaria, Kaduna 810001, Nigeria; aliwazeery@gmail.com; 3 Department of Anatomy, Genetics and Biomedical Informatics, Faculty of Medicine, University of Colombo, Colombo P.O. Box 271, Sri Lanka; hemali@anat.cmb.ac.lk; 4 Implementing Sustainable Evidence-Based Interventions through Engagement (ISEE) Lab, Department of Global and Environmental Health, School of Global Public Health, New York University, New York, NY 10003, USA; ep91@nyu.edu

**Keywords:** β-thalassemia, Sickle-cell disease, pharmacogenomics, pharmacogenetics, DNA polymorphisms, microRNAs

## Abstract

In this short review we have presented and discussed studies on pharmacogenomics (also termed pharmacogenetics) of the drugs employed in the treatment of β-thalassemia or Sickle-cell disease (SCD). This field of investigation is relevant, since it is expected to help clinicians select the appropriate drug and the correct dosage for each patient. We first discussed the search for DNA polymorphisms associated with a high expression of γ-globin genes and identified this using GWAS studies and CRISPR-based gene editing approaches. We then presented validated DNA polymorphisms associated with a high HbF production (including, but not limited to the *HBG2* XmnI polymorphism and those related to the *BCL11A*, *MYB*, *KLF-1*, and *LYAR* genes). The expression of microRNAs involved in the regulation of γ-globin genes was also presented in the context of pharmacomiRNomics. Then, the pharmacogenomics of validated fetal hemoglobin inducers (hydroxyurea, butyrate and butyrate analogues, thalidomide, and sirolimus), of iron chelators, and of analgesics in the pain management of SCD patients were considered. Finally, we discuss current clinical trials, as well as international research networks focusing on clinical issues related to pharmacogenomics in hematological diseases.

## 1. Introduction

**Figure 1:**
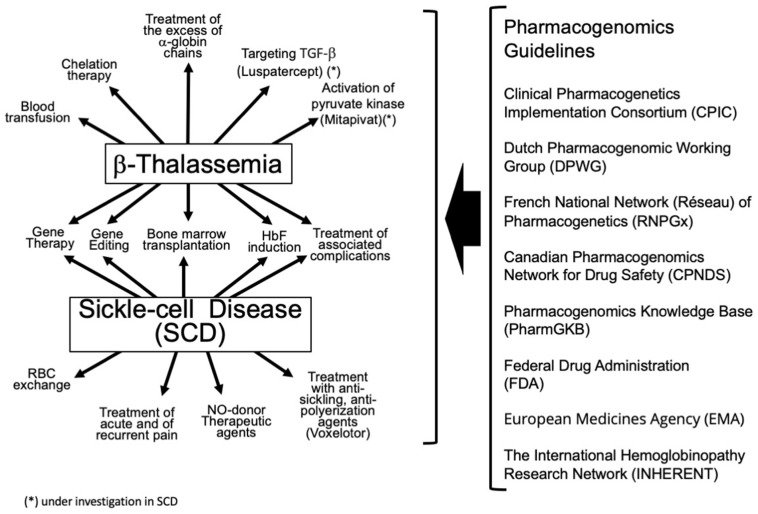
Possible interactions between strategies commonly used and/or proposed for the therapeutic intervention on β-thalassemia and SCD and organizations/networks aiming to facilitate the translation of pharmacogenomics-based information into actionable prescribing decisions for the drugs/agents employed in their treatment. (*) Luspatercept and Mitapivat are under investigation in SCD.

β-thalassemias and Sickle-cell disease (SCD) are the most important inherited genetic disorders affecting the hematopoietic system [1,2]. The phenotype of β-thalassemia is primarily characterized by a low or absent production of adult hemoglobin (HbA) due to low or absent production of β-globin, leading to an excess of free α-globin chains with associated ineffective erythropoiesis [1]. This β-thalassemia phenotype is caused by more than 350 mutations of the adult β-globin gene [1,3,4,5]. The phenotype of SCD is characterized by the presence of a mutated β-globin chain with the replacement (due to a mis-sense point mutation of the β-globin gene) of glutamic acid with valine in the sixth position of the protein, originating the so-called Sickle hemoglobin (HbS) [6]. In hypoxic conditions, HbS polymerizes and interacts with the cytoskeleton, causing the abnormal/Sickle-shaped morphology of red blood cells [7]. Relevant to this review, it should be noted that β-thalassemias and SCD usually require lifelong treatments, some of which are similar (for instance, the induction of fetal hemoglobin -HbF) [8]. In this context, it is very important to consider that, in addition to the primary mutation(s) causing β-thalassemias and SCD, they also exhibit single nucleotide polymorphisms (SNPs) in the genome that can predict the response to the high number of therapeutic treatments that these patients need to face during their lifetime [9]. In this respect, the activity of international organizations devoted to facilitate the translation of the use of these genomic SNPs’ testing into actionable prescribing decisions for related drugs should be monitored [10,11,12]. One example of such an organization is the Clinical Pharmacogenetics Implementation Consortium (CPIC) [12]. In Figure 1, the interplay between the therapeutic strategies for β-thalassemia and SCD and these organizations is depicted.

Pharmacogenomics (also termed pharmacogenetics) [13,14,15] and pharmacoepigenomics [16,17] are fields of applied research that study how the genome and the epigenome affect the response of patients (diagnosed with diseases including β-thalassemia and SCD) to pharmacologic treatment [13,18,19]. Pharmacogenomics studies include (but are not limited to) how variation of the human genome affects differential drug responses [16], whereas pharmacoepigenomics studies deal with the influence that epigenetic alterations (including DNA methylation, histone modifications, chromatin remodeling, and altered expression of non-coding RNAs) exert on drug efficacy and safety [17]. Moreover, pharmacogenomics and pharmacoepigenomics studies also include the effects that drug treatments may have on the genome and or the epigenetic machinery [16,17]. This review is mainly focused on pharmacogenomics and only a few issues (for instance, concerning the impact of non-coding RNAs) related to pharmacoepigenomics have been considered.

The aim of pharmacogenomic research is to achieve optimum care for each individual; thus, pharmacogenomics research helps clinicians select the appropriate drug and the correct dosage for each patient [18,19,20]. Pharmacogenomics can play an important role in differentiating, even among large cohorts of patients, those who are responders and non-responders to a given drug, avoiding adverse events, by selecting the most suitable drug dosage (e.g., genotype-specific dosing) [21]. The variability of biomarkers among patients is a factor to be considered to obtain, for single protocols, the highest probability to reach positive effects on the highest number of treated patients [22]. This research activity involves interdisciplinary collaborations between researchers working in genetics, molecular biology, pharmacology, pharmaceutics, and organic chemistry and it is expected to discover useful information on the mechanisms of drug action (e.g., pharmacodynamics and pharmacokinetics), the identification of novel molecular targets, and the design of novel biomedicines [3,4,5]. Furthermore, the clinical application for the prediction of toxicity risk or beneficial effects could be helpful in numerous ways for several pathologies, including hemoglobinopathies [13,20,23,24,25,26,27].

This aspect of the “Pharmacogenomics” endeavor has a clear impact on personalized treatments in precision medicine for patients carrying β-hemoglobinopathies, such as β-thalassemia and SCD [25,26,27,28,29,30]. In fact, with the help of pharmacogenomics studies, the administration of drugs and drug combinations could be optimized on the basis of the genetic characteristics of each individual [25,26,27].

**Figure 2:**
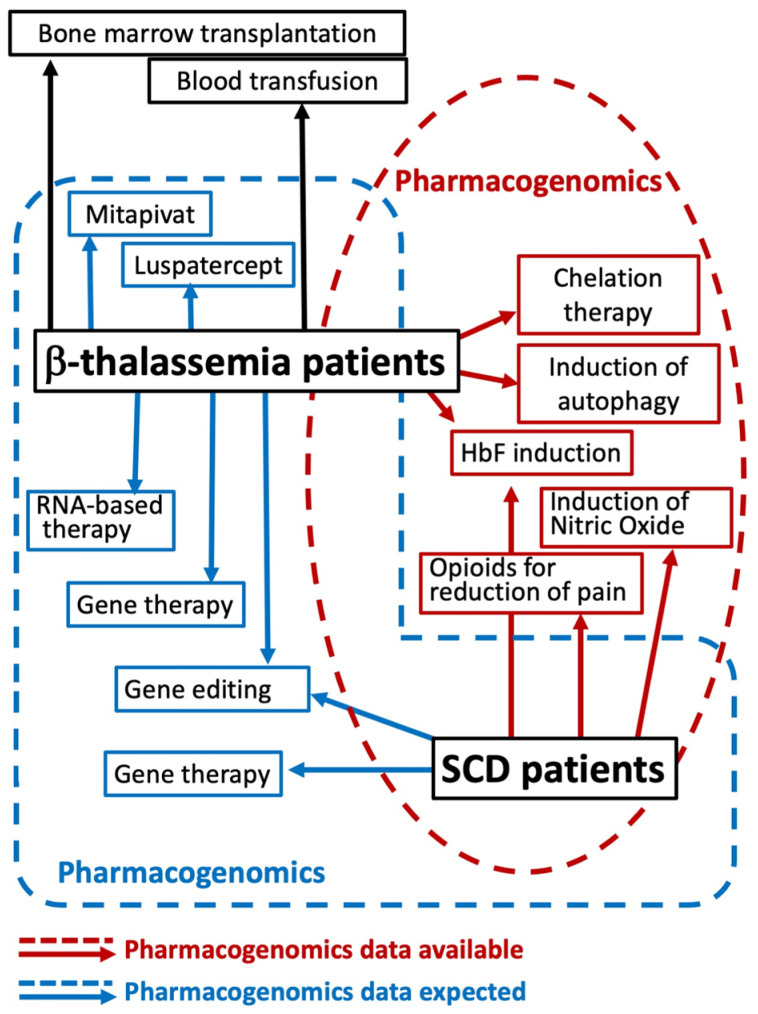
Different therapeutic options for β-thalassemia and SCD in the context of available pharmacogenomics-based studies (most of which are considered in the present review). Red arrows and boxes indicate therapeutic options for which pharmacogenomics information is already available. Blue arrows and boxes indicate therapeutic options for which pharmacogenomics information is expected in the near future.

On the other hand, the changes in the “omics” profile (for instance global methylation, global gene expression, and/or microRNA overall expression), following treatment with drugs employed for the therapy of β-thalassemia, might help in developing further protocols when considered together with the genetic and functional background affecting drug response [31,32,33]. In Figure 2, different therapeutic options for β-thalassemia and SCD are considered for which pharmacogenomics information is already available or is expected in the near future.

**Figure 3:**
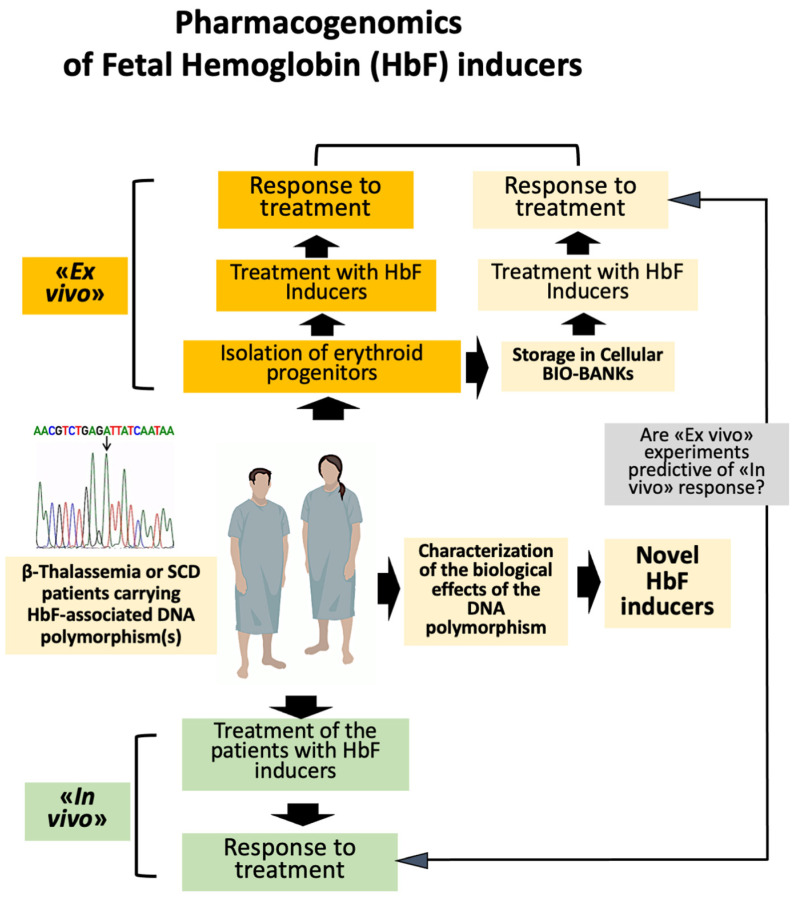
Flow chart of the evaluation of the effects of DNA polymorphism(s) on the response of β-thalassemia patients to an HbF inducer. In the ex vivo protocols, the objective is to verify whether cells isolated from the patient(s) respond to in vitro induction (i.e., increased γ-globin gene expression and HbF production following treatment). The in vivo protocols are based on the treatment of β-thalassemia patients with the HbF inducer (endpoint: reduction, or elimination, of red blood transfusions). In the case of a correlation existing between ex vivo and in vivo protocols, ex vivo assays are predictive of in vivo response.

A simple flowchart summarizing the key points of the pharmacogenomics of HbF inducers is shown in Figure 3.

In this context, two fields of investigation have been highly impactful in understanding the mechanism of action of drugs for β-thalassemia and SCD, as well as the patient-to-patient variation in the response to treatment, as follows: (a) the identification of DNA polymorphisms that predict the response to drug treatment [34] and (b) the post-transcriptional regulation of globin gene expression by non-coding RNAs, including microRNAs [35,36,37]. In vitro, ex vivo, and in vivo studies were deemed to be necessary for a comprehensive understanding of the impact of pharmacogenomics in the management of β-thalassemia patients [38,39,40,41,42,43,44]. In particular, the field of RNA therapeutics (including microRNA therapeutics) should be considered with great attention, because some products from this research field have already reached clinical applications. For instance, inclisiran, a chemically synthesized small interfering RNA targeting PCSK9 messenger RNA [45], has been shown to reduce low-density lipoprotein (LDL) cholesterol levels in clinical trials [46,47]. Interestingly, and of relevance in the context of this review, cardiovascular complications and risk of death are well-known in clinical issues in SCD [48,49]. In this short review, we will first focus on the pharmacogenomics of inducers of fetal hemoglobin (HbF) in erythroid cells from β-thalassemia patients. The increased expression of γ-globin genes and a high HbF induction in β-thalassemia patients are associated with a milder or even asymptomatic disease pattern [50,51,52]. In this case, a transfusion regimen and chelation therapy might not be necessary. In addition to β-thalassemia, the high-level production of HbF could be highly beneficial for patients with SCD [53,54]. Despite the fact that the clinical phenotypes in SCD are considerably heterogeneous, a reduction in pain episodes and avascular necrosis of the femoral head (that are both common clinical features of SCD) are reduced in SCD patients exhibiting high levels of HbF.

In consideration of the role of HbF production in β-thalassemia and SCD, efficient HbF inducers were developed for the treatment of β-thalassemia patients characterized by a low production of HbF [55,56,57,58,59]. Despite the high and growing numbers of HbF inducers, clinical trials are ongoing for a limited number of them, as depicted in Table 1 [60,61,62,63,64,65,66,67,68,69,70,71,72,73,74,75,76,77,78,79,80,81,82].

## 2. The Search of DNA Polymorphisms Associated with High Expression of γ-Globin Genes: GWAS Studies and CRISPR-Based Gene Editing Approaches

Historically, the most important contribution to the identification of DNA polymorphisms associated with high HbF production came from genome-wide association studies (GWAS) [83,84,85,86,87,88,89]. For instance, GWAS were a key approach to identify BCL11A as a candidate repressor of the expression of γ-globin genes [85]. In addition, GWAS-identified single nucleotide polymorphisms (SNPs) have been mapped within the erythroid enhancer of the *BCL11A* gene [84]. This was a key contribution to the development of the pharmacogenomics of β-thalassemia, especially after the development of gene editing protocols that were able to demonstrate that the disruption of discrete portions of the *BCL11A* enhancer are associated with a reduction in the expression of BCL11A and an increase in γ-globin gene expression and HbF production [90,91,92]. More recently, Antoniou et al. have used base editing to generate a large variety of mutations within the −200 region of the γ-globin gene promoters [91]. Most of the mutations were found to deeply alter the binding of transcriptional repressors to the promoter. Editing of hematopoietic stem/precursor cells HSPC isolated from patients was able to induce HbF reactivation, rescuing the pathological phenotype [91]. Therefore, β-thalassemia patients carrying polymorphic DNA sequences causing these clinically relevant effects are expected to respond to some Hb inducers with a high efficiency. This can be studied both ex vivo (using erythroid progenitor cells isolated from β-thalassemia or SCD patients) and in vivo (treating β-thalassemia or SCD patients with the HbF inducer and verifying their response to the therapeutic intervention) (see Figure 3).

**Figure 4:**
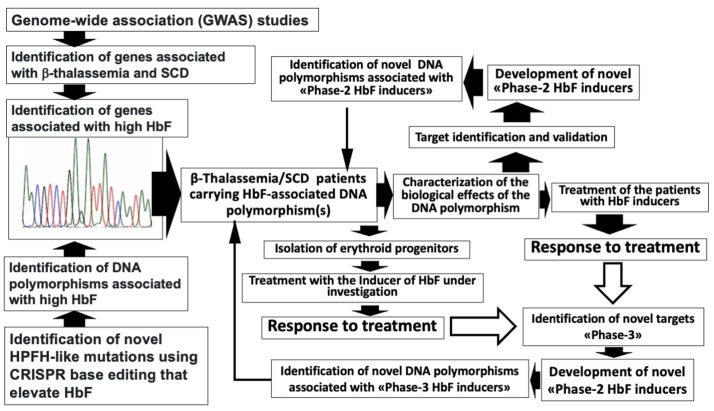
The interplay between GWAS approaches, gene editing, and pharmacogenomics. These combined approaches can ultimately help in identifying novel targets to be modulated with novel drugs to be further studied with pharmacogenomic protocols. (HPFH: hereditary persistence of fetal haemoglobin; HbF: fetal hemoglobin; CRISPR: Clustered regularly interspaced palindromic repeats.

The interplay between GWAS, gene editing, and pharmacogenomics is further described in Figure 4, which also underlines that fully integrated procedures might help in the identification of novel targets, in the development of novel drugs, and, consequently, in the identification and characterization of novel DNA polymorphisms.

## 3. Major DNA Polymorphisms Associated with High Expression of γ-Globin Genes

Several reports concurrently demonstrate that the response to HbF inducers might depend on HbF-associated polymorphisms (e.g., *HBG2* XmnI, *BCL11A*, and *MYB* polymorphisms) [93,94], and patient stratification based on these genetic characteristics might be useful for clinical management and the choice of the therapeutic protocol. For instance, Roy et al. studied the influence of *BCL11A*, *HBS1L-MYB*, and *HBBP1* single nucleotide polymorphisms (in addition to the *HBG2* XmnI polymorphism) on the levels of endogenous production of HbF and found a significant association of HbF levels with rs2071348 (*BCL11A*) and rs4895441 (*HBS1L-MYB*), respectively. The *HBG2* XmnI polymorphism showed the strongest association [76,77,78,79,80,81,82,83,84,85,86,87,88,89,90,91,92,93,94,95]. An extensive description of SNPs associated with high production of HbF is outside the objectives of the present review that is mainly focused on the most relevant (in our opinion) DNA polymorphisms considered in published pharmacogenomic studies on β-thalassemia and SCD. However, important studies and review articles on DNA polymorphisms associated with the high expression of γ-globin genes and HbF production are available in the literature, such as (among others) those published by Pace B and Zein [96], Biswas et al. [34], Stadhouders et al. [97], Patrinos et al. [25], Karamperis et al. [26] and Mnika et al. [28]. 

### 3.1. The HBG2 XmnI rs7482144 Polymorphism

The *HBG2* XmnI polymorphism is among the most studied and characterized genetic variants. Furthermore, this polymorphism was found to be strongly associated with HbF levels in β-thalassemia patients and with the response to HbF inducers [98]. For instance, Tien Nguyen et al. found that in β-thalassemia intermedia patients with various genotypes, the XmnI (G)gamma polymorphism at position −158 of the *HBG2* promoter displayed a deep effect on fetal hemoglobin levels [99]. Furthermore, the XmnI rs7482144 polymorphism helps in the genetic prediction for age at onset and life expectancy of β-thalassemia patients [100]. In addition, this polymorphism has been associated with high levels of HbF in Sickle-cell anemia [101,102].

### 3.2. The LYAR rs368698783 (G>A) Polymorphism: Relevance and Structural Association with HBG2 XmnI

Recently, Ju et al. [103] identified a putative novel regulator of γ-globin gene transcription, LYAR (human homologue of mouse Ly-1 antibody reactive clone). The LYAR DNA-binding motif (5′-GGTTAT-3′) was identified in a DNA region corresponding to the 5′-untranslated region of the Aγ-globin gene. LYAR is a strong repressor of human γ-globin gene transcription, suggesting this is a novel transcription factor essential for γ-globin gene silencing [104]. Interestingly, Bianchi et al. [105] and Breveglieri et al. [106] found that the rs368698783 (G>A) polymorphism is present in β-thalassemia patients [105,106]. Moreover, molecular docking simulations, the electrophoretic mobility shift assay (EMSA), and Biacore analyses demonstrated that the rs368698783 (G>A) polymorphism decreases the LYAR binding efficiency to the Aγ-globin gene, confirming that it might affect cellular responses to HbF inducers [107]. Using rapamycin as an HbF inducer and erythroid precursor cells (ErPCs) isolated from β-thalassemia patients as an experimental model system, Zuccato et al. found that rs368698783 (G>A) is associated with a high induction of HbF [108].

### 3.3. The BCL11A rs2071348 Polymorphisms

The rs2071348 polymorphisms, together with the *HBG2* XmnI rs7482144 polymorphism, help in the genetic prediction for age at onset and life expectancy [79]. Interestingly, Giannopoulou et al., studying a group of β-thalassemia major (β-TM) patients (severe phenotype) and β- thalassemia intermedia (β-TI) patients (mild phenotype), found that the rs2071348 polymorphism was associated with higher HbF levels and a milder β- thalassemia disease phenotype [109].

### 3.4. The KLF1 rs3817621, rs2072597, and rs2072596 Polymorphisms

The erythroid-specific Krupple-like factor 1(KLF1) is a transcription factor that regulates the beta-like globin gene expression, and variations within the gene can alter the SCD phenotype [110]. KLF1 is the master regulator of the *BCL11A* gene and also mediates hemoglobin switching [111,112]. Of the three novel SNPs (rs3817621, rs2072597, and rs2072596) identified in 118 SCD patients in central India, no statistically significant association of any of the SNPs identified in the polymorphisms was seen with HbF levels, as well as the SCD-related morbidities [111]. However, rs3817621 was reported in association with fetal hemoglobin in Thai hemoglobin E (HbE)/E β-thalassemia [113] and HbF in β-thalassemia carriers and normal individuals of Portuguese origin [114]. Although rs2072597 was not found to be associated with SCD patients in central India, the same SNP was found to be associated with HbE/β-thalassemia [95]. Finally, rs2072596 is associated with high HbA2 levels in Iranian thalassemia patients [115], but not in patients from Saudi Arabia [116].

### 3.5. Other Genetic Polymorphisms

Other genetic variants have been studied and their characterization has been included in several reports. For instance, genomic variants of the *MAP3K5*, *NOS2A*, and *ARG2* genes were associated with hydroxyurea (HU) therapy efficacy [96]. In the same study, it was also reported that the *FLT1* and *ARG2* genomic variants were associated with a mild phenotype of NTDT patients. The findings reported by Kolliopoulou et al. [117] provide evidence that the *MAP3K5*, *NOS2A*, *ARG2*, and *FLT1* genomic variants could be considered as genomic biomarkers to predict HU therapy efficacy in Hb S-β-thalassemia compound heterozygotes and also to describe disease severity in patients with β-type hemoglobinopathies [117].

## 4. Expression of microRNAs Involved in the Regulation of γ-Globin Genes: Evidence Supporting PharmacomiRNomics

### 4.1. Non-Coding RNAs and HbF Production

Non-coding RNAs (ncRNAs) are important regulators of gene expression in eukaryotes [118,119] and have been demonstrated to be involved in β-thalassemia [120,121,122] and SCD [123,124]. This is a rapidly evolving field of investigation. The most studied class of non-coding RNAs are microRNAs, a class of short (less than 30 nucleotides in length) ncRNAs with a very important role in the regulation of gene expression at the post-transcriptional level [125,126,127] through sequence-specific interactions with the 3′UTR of target mRNAs. This causes translational repression or mRNA degradation [126]. Several miRNA/mRNA networks are responsible for the control of important biological functions, such as cell growth, apoptosis, and differentiation [127], and their alterations are associated with the onset and/or progression of several human pathologies [128,129,130].

In the context of RNA- and DNA-based therapeutics, including microRNA therapeutics, the issue of delivery should be mentioned, since it is or paramount relevance in bringing laboratory research to the clinic, greatly facilitating the approval of therapeutic protocols by the regulatory agencies, such as the European Medicines Agency (EMA) in Europe and the Food and Drug Administration (FDA) in theUS [131]. This issue has been considered in excellent reviews [131,132,133]. In these review articles, polymeric nanoparticulate delivery systems are extensively described, such as lipid-based nanoparticles, polymeric micelles, dendrimers, and polymer–drug conjugates [131,132,133,134,135,136]. These systems offer stability, protection, and controlled release [131,133]. In addition, they are expected to be ready for clinical applications when important traits are fully developed, such as the consistent bioactivity across batches; the possibility to be re-dosable, without changes in efficacy and safety; the NHP safety at doses greater than those required for efficacy; acceptable on-/off-target delivery profile in NHPs; and the possibility to be manufactured at the human scale, with a scalable and biodegradable chemistry. These features of delivery systems have been fully considered by Paunovskaet al. [131]. An example is constituted by the Phase 1 study of MRX34, a liposomal miR-34a mimic [137]. This is of great interest in the context of our study, as the stable expression of miR-34a was found to mediate HbF induction, possibly involving STAT3 gene silencing [138].

### 4.2. MicroRNAs and HbF Production

The role of non-coding RNAs (including microRNAs and miRNAs) in HbF regulation in β-thalassemia and SCD has recently been investigated by many research groups [35,36,37,139,140,141,142,143,144,145,146,147,148,149,150,151]. Concerning microRNAs, Kargutkar et al. investigated the possible relationship between the expression of miRNAs and the induction of HbF in subjects (including SCD patients) treated for 3 and 6 months with hydroxyurea [35]. A differential expression of miRNAs during treatment with HU was found and was associated with the HU-mediated increase in HbF. This was particularly evident for the increase in miR-210, miR16-1, and miR-29a expression and the decrease in miR-96. As this study strongly suggests that miR-210, miR-16-1, and miR-29a are positive regulators of γ-globin gene expression and miR-96 is a negative regulator, the pattern of expression of miRNAs should be considered in further studies aimed at verifying the possibility to predict response to HU, based on the miRNome of each patient. A study by Kargutkar et al. confirms a possible role in HbF induction of miR-210, which was proposed by Gasparello et al. to target BCL11A, the major repressor of γ-globin gene expression [143]. Other examples of microRNAs involved in the regulation of γ-globin genes are miR-92a-3p [144] and miR-30a [145]. A partial list of the non-coding RNAs involved in the regulation of γ-globin genes is reported in Table 2 [143,144,145,146,147,148,149,150,151,152,153,154,155,156,157,158].

All the information available supports the concept that non-coding RNAs, including microRNAs (miRNAs), long non-coding RNAs (lncRNAs), and circular RNAs (circRNAs), are deeply involved in the regulation of γ-globin gene expression [139,140,141,142,143,144,145,146,147,148,149,150,151,152,153,154,155,156,157,158,159]. Exploring possible crosstalk between long non-coding RNAs and microRNAs will clarify their possible role as prognostic and therapeutic biomarkers in β-thalassemia [160]. Interestingly, most of the miRNA targets are mRNA coding transcription factors, regulating γ-globin gene expression [143,144,145,146,147,148,149,150,151,152,153,154,155,156,157,158,161]. The impact of non-coding RNAs in the prediction of response to HbF inducers has lead to several observations and/or comments supporting the concept of “PharmacomiRNomics” of HbF inducers (Figure 3), as already proposed by Faraoni et al. (2009) [162] in a study focusing on the variability of miR-155 expression profiles in respect to physiological and pathological processes [162]. Das et al. [163] were able to identify SNPs in pre-miRNA regions and pre-miRNA flanking regions using β-thalassemia as a model system. Most of these SNPs were expected to alter the translation process of miRNA-bound mRNAs, thereby altering HbF production, with an impact on the clinical severity of the disease [163]. Micro-RNAs miR-486 and miR-26b had the highest number of SNPs in upstream and downstream flanking regions, respectively [27]. DNA polymorphisms within the 3′UTR of target mRNAs coding for proteins regulating γ-globin gene expression (for instance BCL11A, MYB, SOX-6, and KLF-1) have also been identified. Furthermore, polymorphisms of the binding sites of microRNAs regulating γ-globin gene expression have also been found [163]. These data further sustain the concept, based on microarray and NGS studies, that a sub-set of microRNAs is associated with the HbF expression levels [159,160,161].

**Figure 5:**
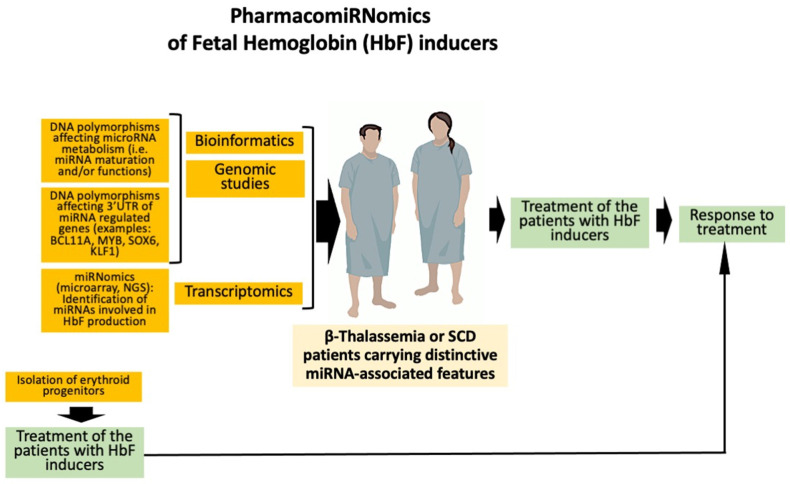
The basic steps of “PharmacomiRNomics” of HbF inducers in β-thalassemia and SCD.

The impact of these data on the possible proposal of “PharmacomiRNomics” (Figure 5) is sustained by two recently published studies. In the first, Hojjati et al. studied β-thalassemia patients treated with HU and grouped them into two groups, as follows: responders and non-responders to the treatment [164]. The results obtained demonstrated that miR-210 and miR-486-3p were expressed at higher levels in the responders than in the non-responders group.

More recently, Kargutkar et al. evaluated the effect of miRNA expression on HbF induction in relation to HU therapy in normal controls, SCD patients [35]. Among the most relevant findings of this study, an increase in miR-210, miR16-1, and miR-29a expression and a decrease in miR-96 expression were strongly associated with the HU-mediated HbF induction [35].

While the results of these studies are promising, further investigations will be necessary to verify the real impact of “PharmacomiRNomics” in the management of the therapeutic options for β-thalassemia and SCD patients, with a particular focus on HbF induction.

## 5. Pharmacogenomics of Fetal Hemoglobin Inducers in β-Thalassemia and Sickle-Cell Disease: Ex Vivo and In Vivo Studies

### 5.1. Hydroxyurea

Changes in HbF levels upon HU therapy are likely to be regulated by multiple loci in both β-thalassemia and SCD [165]. The identification and characterization of these SNPs are important, as this improves the clinical management of the patients [25,166]. In the case of HU, several studies indicate a relationship between the *HBG2* XmnI polymorphism and the induction of HbF, as reported in different studies [165,166,167,168]; these studies are expected to stimulate further analyses of the degree of response of transfusion-dependent β-thalassemia patients to HU therapy in order to develop a shared diagnostic protocol to identify patients likely to have a significant response [169]. In this respect, Gosh et al. [170]. found that HU therapy conducted in HbE/β-thalassemia-major patients was able to improve the transfusion-free interval and was correlated with the *HBG2* XmnI polymorphism [170]. Other SNPs associated with HbF induction should be considered in the development of a final diagnostic protocol for the prediction of HU response in β-thalassemia and SCD. In this respect, Giannopoulou et al. reported that the rs2071348 polymorphism in the *HBBP1* gene does not correlate with response to HU treatment [109]. Relevant to this topic is a systematic review focused on seven studies involving SCD patients treated with HU; 50 SNPs of 17 different genes were identified and found to be associated with HbF changes following HU monotherapy (Sales et al., 2021) [166]. The authors confirmed that HU-mediated changes in HbF levels in response to HU therapy are regulated by genetic variations on multiple loci [166]. A second conclusion gathered from the studies reviewed by Sales et al. is that in those studies, there was strong evidence that SNPs located in intron 2 of the *BCL11A* gene affect HbF changes in SCD patients treated with HU [166]. In conclusion, in spite of the variability of clinical protocols and the heterogeneity of patients studied, the available information strongly supports the concept that pharmacogenomics studies on HbF induction in response to HU are of potential application in improving the clinical management of β-thalassemia and SCD.

### 5.2. Butyrate and Butyrate Analogues

Butyrates are a class of histone deacetylase (HDAC) inhibitors demonstrated to be able to increase γ-globin gene expression and HbF content in erythroid cells from β-thalassemic and SCD patients [171,172,173]. It has been reported that at least 25% of the patients affected by hemoglobinopathies do not respond to treatment with butyrates [174,175]. However, despite the fact that butyrates have been proposed as good candidate drugs for pharmacogenomic studies [174], few reports have been published on this issue. An interesting study is based on the observation that patients who are unresponsive to HU treatment can be treated with sodium butyrate containing microRNAs to induce HbF synthesis [174,176]. 

### 5.3. Thalidomide

A meta-analysis has recently been published on the efficacy of thalidomide in the induction of HbF in thalidomide-treated β-thalassemia patients [177]. This study was unable to detect any statistically significant relationships between the efficacy of thalidomide in transfusion-dependent β-thalassemia patients and the XmnI polymorphism [177]. In another study, Yang et al. considered the relevance of *HBG2* (rs7482144), *BCL11A* (rs11886868, rs4671393, rs766432, and rs1427407), and *HBS1L-MYB* (rs9399137, rs4895440, and rs4895441) single nucleotide polymorphisms (SNPs) in thalidomide response [178]. This study demonstrated that SNPs in *HBG2* and *HBS1L-MYB* contributed significantly to thalidomide response in Chinese patients with β-thalassemia and that the cumulative number of minor SNP alleles may serve as good predictors of treatment response in this population [178]. In conclusion, pharmacogenomics of HbF induction in response to thalidomide should be considered in further studies on β-thalassemia and SCD patients in order to confirm the contribution of the identified SNPs in *HBG2* and *HBS1L-MYB* in the response to thalidomide and the hypothesis of a lack of correlation between response to thalidomide and the *HBG2* XmnI polymorphism.

### 5.4. Rapamycin (Sirolimus)

Recently, Zuccato et al. reported a study demonstrating that the *LYAR* rs368698783 (G>A) polymorphism is associated with a high basal and induced production of HbF in β-thalassemia patients [108]. The most striking association was found using rapamycin (sirolimus) as an HbF inducer. The published results are important not only for basic biomedicine, but also in applied translational research for precision medicine in the personalized therapy of β-thalassemia [108]. Accordingly, the rs368698783 polymorphism might be considered among the parameters useful to recruit patients with the highest probability of responding to in vivo rapamycin treatment.

## 6. Pharmacogenomics of Iron Chelators in β-Thalassemia and Other Hematopoietic Diseases Needing Blood Transfusions

As extensively discussed in several review papers, the management of β-thalassemia patients needing blood transfusion (TDT, transfusion-dependent thalassemia) is based on the use of an iron-chelation therapy in order to eliminate the iron excess associated with blood transfusion. Pharmacogenomics can greatly help clinicians to identify the best iron chelator to be used, considering a number of well-defined genetic polymorphisms, as discussed in the following examples, concerning the two most studied iron chelators, Deferasirox and Deferiprone.

### 6.1. Deferasirox

Deferasirox (DFX) is an oral iron chelator for the management of chronic iron overload in patients with chronic anemias who require blood transfusions [179]. The available literature suggests the safety and effectiveness of DFX, but a rather heterogenous response in treating β-thalassemia patients [180,181]. The efficacy of DFX in other chronic transfusion-requiring diseases is, at present, extensively studied with promising results. Single nucleotide polymorphisms in genes involved in DFX metabolism (such as *UGT1A1*, *UGT1A3*, *CYP1A1*, *CYP1A2*, and *CYP2D6*) and elimination (such as *MRP2* and *BCRP1*) have been investigated, in order to determine whether some of them were found to be associated with DFX plasma concentration and, consequently, the response of the patients to DFX. The most studied polymorphisms are the *UGT1A1*C>T rs887829, *CYP1A1*C>A rs2606345, *CYP1A2A*>C rs762551, *CYP1A2*C>T rs2470890, and *MRP2G*>A rs2273697 polymorphisms. The *CYP1A1*C>A rs2606345 AA and *CYP1A2C*>T rs2470890 TT genotypes were found to be useful in the prediction of low response of the patients to DFX therapy [182,183].

### 6.2. Deferiprone

Deferiprone is a bidentate oral iron chelator. Deferiprone is metabolized into deferiprone-3-0-glucuronide (DG) by the enzyme UDP-glucuronosyl transferase (UGT). *UGT* belongs to four gene families, *UGT1*, *UGT2, UGT3* and *UGT8* [184,185]—Among several isoforms of UDPglucuronosyltransferases, UGT1A, UGT2B, and UGT1A9 are responsible for the glucuronidation of deferiprone [184]. Two variants, −2152 C>T and −275 T>A, in the promoter of the *UGT1A9* gene are associated with the increased expression of *UGT IA9*. Therefore, the response to deferiprone-based therapy may be influenced by those genetic variants. It has been known that there are three commonly known polymorphisms in UGT1A6, which are Thr181Ala (541 A>G), Arg 184 Ser (552 A>C), and Ser 7Ala (19 T>G); these are responsible for the efficacy of deferiprone treatment [185].

## 7. Pharmacogenomics of Analgesics in Pain Management of SCD Patients

Pain is the hallmark of SCD, leading to recurrent admissions to the emergency department. The most common way of combating it is through admissions of analgesics such as NSAIDs and opioids and, in some instances, adjuvants. The cytochrome P450 superfamily, in which CYP2C8 and CYP2C9 belong, plays a major role in their metabolism. Both enzymes have been associated with a decreased enzyme activity alteration in their pharmacokinetics [186,187].

More than 16 alleles and over 60 variants have been characterized. These allelic variants impact their metabolic activity leading to classification into ultra-rapid responders, extensive metabolizers, intermediate metabolizers, and poor metabolizers. Most analgesic dosing and pain plans assume patients to be extensive metabolizers (i.e., having two functional alleles). However, sub-optimal responses and adverse side effects have been demonstrated in individuals having varying polymorphism at *CYP2C8* and *CYP2C9* [188]. In the context of analgesics, CPIC guidelines and the FDA drug labels are available [189,190].

### 7.1. Pharmacogenomics of Opioids

Opioids are of interest for the treatment of pain in SCD patients, as they are among the first choices for the management of the patients, with respect to this very important clinical complication. Accordingly, chronic opioid therapy might be, in some cases, considered, especially in the case of SCD patients who are debilitated by chronic pain. The pharmacogenomics of opioids have been considered in a review article by Husain et al. [189], who briefly reviewed the literature on pharmacogenomics in SCD and possible targets for intervention in the context of possible steps on the road to personalized medicine, considering that the SCD patients exhibit a high variability in response to opioids. Polymorphisms in the *COMT*, *OPRM1*, and *ABCB1* genes can lead to an altered perception of pain and/or a change in response to opioids by the SCD patients. This information can be found in the studies reported by Joly et al. [190] and Jhun et al. [191]. Joly’s study is relevant, since it was conducted in a significant cohort of SCD patients [190]. With respect to pharmacokinetics, important SNPs have been genotyped for the *CYP2D6* gene (codeine to morphine conversion) and for three genes involved in morphine elimination (*CYP3A*, *UGT2B7*, and *ABCB1*), but more pharmacokinetic studies on phenotype/genotype correlations are needed to reach definitive conclusions [190]. The pharmacogenomics of morphine have recently been discussed [192]. The role of pharmacogenomics in prescribing opioids has been discussed by Wong et al. [193], discussing the situation concerning cancer patients. In this case, pharmacogenomic evidence guiding opioid prescription is currently available for codeine and tramadol, which relates to the *CYP2D6* gene variants. Moderate evidence has been reported for oxycodone (which relates to *CYP2D6*) and methadone (which relates to *CYP2B6* and *ABCB1*) [193]. The experience in cancer will probably guide the research efforts concerning SCD, as demonstrated by the study by Gammal et al. [194], who discussed the role of pharmacogenomics (in particular focusing on the *CYP2D6* genotype for the safe use of codeine in SCD). This study is particularly relevant, considering the adverse effects of postoperative deaths in children who were prescribed codeine [194].

### 7.2. Inducers of Nitric Oxide in SCD

Nitric oxide (NO) deficiency has been shown to play an important role in the development of vaso-occlusive crises, a characteristic feature of SCD [188]. Accordingly, clinical trials and studies have suggested the importance of artificial NO induction in relation to a reduction in the severity of the disease [195]. In this respect, Weiner et al. [196] reported a preliminary study suggesting that inhaled NO may be beneficial for acute vaso-occlusive crises in SCD [196]. Since endothelial Nitric Oxide Synthase (eNOS) plays a major role in regulating the NO levels in the body, studies have been focused on the polymorphism(s) of the *eNOS* gene, in order to verify whether they can be predictive of the severity of SCD [197]. In this respect, several SNPs of the *eNOS* gene were found in SCD patients to be associated with NO levels and the severity of the disease [198,199]. For example, rs2070744 was found in the promoter region and rs1799983 within exon 7 (894 G>T, Glu-298Asp) and the frequency of these polymorphisms was particularly high in patients with severe SCD, associated with lower levels of plasma NO [199]. NO inducers should be considered as therapeutic tools in SCD [200,201]. Interestingly, evidence for the in vivo conversion of hydroxyurea to nitric oxide has been provided [202].

## 8. Guidelines and Clinical Trials Focusing on Pharmacogenomics/Pharmacogenetics in β-Thalassemia and SCD

Although knowledge of the association of SNPs on pharmacodynamics and pharmacokinetics has increased, as of 2016, the issue of utilizing pharmacogenomics for β-thalassemia and SCD remains contentious. In parallel, several activities are ongoing to help clinicians follow the best possible management of β-thalassemia and SCD patients. For instance, some of the pharmacological interventions that are used to manage SCD have specific guidelines created by the Clinical Pharmacogenetics Implementation Consortium (CPIC) [203]. The CPIC evidence-based guidelines can improve outcomes by utilizing guide dosing [30]. The CPIC publishes genotype-based drug guidelines to help clinicians understand how available genetic test results could be used to optimize drug therapy [203]. Using electronic medical records, Gallaway and colleagues found that CPIC guidelines could have been used during a 16 year (2005 to 2021) period, in which 892 (93%) patients at Indiana University received SCD care [30]. A total of 814 (85.1%) patients received two drugs with CPIC guidelines, 654 (68.3%) received three drugs, 355 (37.1%) received four drugs, 157 (16.4%) received five drugs, 63 (6.6%) received six drugs, 21 (2.2%) received seven drugs, and one (0.1%) received eight drugs [16]. In total, 65 (6.8%) patients received no drugs with CPIC guidelines [8]. Nevertheless, Gallaway and colleagues highlight that SCD patients had a high utilization of drugs with CPIC guidelines [30] and received an average of three unique CPIC drugs/drug classes per person. Table 3 summarizes examples of pharmacogenomics gene–drug association, using information available from the CPIC and FDA concerning drugs employed or proposed for SCA and β-thalassemia therapy [203].

Although many acknowledge that greater progress has been made toward identifying the key genomic variants, mainly in *BCL11A*, *HBS1L-MYB*, or *SAR1*, which contribute to the response to HU treatment [28], some argue that the complete picture of pharmacogenomic/genetic determinants of therapeutic phenotypes remains elusive [28]. Moreover, no large-scale studies have been conducted in sub-Saharan Africa, where the majority of patients with SCD live to elucidate the phenotype/genotype relationships [16]. In contrast, others have suggested tailor-made “theranostics” in SCD as the way forward, able to direct optimal clinical management of the patients [203,204,205,206,207,208].

To assess the current state of clinical trials, we use the US Clinical Trials website. Although this website contains comprehensive information for trials in the US, trials in many countries are not captured in this dataset. After a first search using the ClinicalTrials.gov database (https://www.clinicaltrials.gov, accessed on 25 March 2024), 279 studies were found for β-thalassemia and 972 for SCD. Of these studies, 7–10% (23 studies for β-thalassemia and 72 for SCD) indicated HbF production as an endpoint. When drugs, here reviewed, were considered, most of the studies (15 studies for β-thalassemia and 172 for SCD) were based on HU. No study was primarily focused on “pharmacogenomics in β-thalassemia or SCD”. We can conclude that the design and activation of this field of clinical investigation is still in a preliminary phase. 

In the current landscape of trials, 199 trials match the “sickle cell” text search in the ClinicalTrials.gov database that are currently recruiting participants; of those, 44 trials are industry funded, with other trials being a combination of government, academia, and other organizations. The only ongoing clinical trial that matched the search terms “Pharmacogenomic” and “Sickle Cell” in the NIH Clinical Trials database (conducted in June 2023) was the Therapeutic Response Evaluation and Adherence Trial (TREAT). No trials matched the search terms “Pharmacogenetic” and “Sickle Cell”. The TREAT clinical trial is funded by the US NIH and located at Cincinnati Children’s Hospital Medical Center and has the following objectives: (1) to develop and prospectively evaluate a population pharmacokinetic/pharmacodynamics model to predict the maximum tolerated dose (MTD); (2) to identify urine biomarkers of HU adherence using a novel metabolomics approach; (3) to identify pharmacogenomics modifiers of HU MTD; and (4) longitudinal monitoring of the effect of HU upon organ function and quality of life (https://clinicaltrials.gov/ct2/show/NCT02286154, accessed on 25 March 2024) and recruitment is still ongoing. The TREAT recruitment cohort consists of individuals aged from 6 months to 21 years, diagnosed with SCA (HbSS or Hbβ0-thalassemia) and will consist of patients who initiate HU therapy, including patients who are transitioning from chronic transfusions to HU therapy. The enrolled participants will receive HU; however, upon enrollment, participants will be grouped as a “New Cohort” or “Old Cohort”. “New Cohort” participants include those who are not receiving HU therapy upon study entry; for group pharmacodynamics and pharmacokinetics, data will be used to predict the most effective maximum tolerated dose. “Old Cohort” participants include those who are already receiving HU therapy and participants will receive HU escalated to MTD based on local clinical guidelines.

A search of the NIH trials database indicates that currently, 40 clinical trials match the search terms “hydroxyurea” and “sickle cell” with currently 33 recruiting and 7 designated as not yet recruiting. Among clinical trials utilizing a pharmacogenomics/genetics approach, HU is the most studied pharmacological modifier [166]. Sales and colleagues utilized a systematic review and found 50 different SNPs of 17 genes to be associated with HbF changes in patients with SCD treated with hydroxyurea that are VEGF ligand–receptor interactions (R-HSA-194313; R-HSA195399) and the urea cycle(R-HSA-70635) [166]. Further, Sales and colleagues concluded from their systematic review that pharmacogenetic studies in patients with SCD were sparse and very heterogeneous in regard to candidate genes and SNPs examined for the association with HbF changes and outcomes, suggesting further research is needed [166].

## 9. Conclusions and Future Perspectives

In this short review, we have presented and discussed studies on pharmacogenomics and the response of patients (diagnosed with diseases including β-thalassemia and SCD) to pharmacologic treatment.

The issue of pharmacogenomics in hematological diseases has been considered in several concluded European Union-funded research projects, as well as in ongoing initiatives. For instance, the study of the association between DNA polymorphisms and phenotypes in β-thalassemia was considered a key issue in the ITHANET (Electronic Infrastructure for Thalassemia Research Network, FP6) [209] and THALAMOSS (Thalassemia Modular Stratification System for personalized therapy of β-thalassemia, FP7) [210] EU-funded projects. Furthermore, this field of investigation is central in two ongoing projects related to hemoglobinopathies (such as thalassemia and SCD), the COST-action CA22119–HELIOS and the INHERENT (The International Hemoglobinopathy Research Network) projects.

INHERENT is a recently established network with the aim of investigating the role of genetic modifiers in hemoglobinopathies, through a large-scale, multi-ethnic genome-wide association study (GWAS). INHERENT brings together nine existing international or regional consortia in the field of hemoglobinopathies, namely ITHANET, RADeep [211], ARISE, SPARCO, SADaCC, REDAC, the HVP Global Globin Network [212], the International Health Repository, and the ClinGen Hemoglobinopathy VCEP [213].

In particular, we expect that the large INHERENT research group will be able to sustain research efforts on the discovery/validation of known and new genetic modifiers for the pharmacogenomics of HbF inducers, as well as for other drugs employed in the management of β-thalassemia and SCD patients. In fact, the identification and characterization of a few known genetic modifiers (including those reviewed in the present study) are, as yet, insufficient to guide treatment recommendations or to reliably stratify patients. As proposed in the review article by Kountouris et al. [214], larger multi-ethnic studies are needed to identify and validate further disease modifiers that can be used for patient stratification and personalized treatment. The INHERENT network is expected to greatly help in these research efforts.

In addition, future perspectives include the use of multiple genetic modifiers to verify the response of the patients to drugs to be considered in therapy. Furthermore, we have to comment on the fact that several drugs/molecules for β-thalassemia and SCD have recently reached the market and/or are, at present, being tested in clinical trials. For most of them (for instance, mitapivat, luspatercept, and the gene editing drug CTX001), pharmacogenomic studies are not available yet (see Figure 2). Finally, up to now, most pharmacogenomic-oriented studies have been performed using single-drug treatments. In the future, we expect that combined treatments will be tested in order to reach the highest benefit possible for β-thalassemia and SCD patients. These new protocols will be considered for pharmacogenomic analyses.

A final comment is related to molecular diagnosis and, in particular, to non-invasive prenatal diagnosis (NIPD). In this field of investigation, several approaches have been tested on β-thalassemia, with the aim of identifying the primary mutations [155]. In this respect, the discovery of cell-free fetal DNA (cffDNA) in the peripheral blood of pregnant women has encouraged the research of innovative molecular protocols providing the possibility of NIPD [215]. For instance, the possibility of NIPD has been demonstrated using multiplex PCR/NGS [216], pyrosequencing [217], and droplet digital PCR [218,219,220]. In the near future, we expect that these protocols will be applied to the non-invasive prenatal identification of DNA polymorphisms associated with better outcomes of clinical treatments in β-thalassemia and SCD.

## Figures and Tables

**Table 1:** Fetal hemoglobin inducers and ongoing clinical trials.

Agent	Comments	References
5-Azacytidine	DNA hypomethylation	Kalantri et al., 2018 [60]
Hydroxyurea	DNA hypomethylation	Fucharoen et al., 1996 [61]
Butyrate	HDAC activity inhibition	Bianchi et al., 2015 [62]
2,2-dimethylbutyrate (HQK-1001)	A phase II study of efficacy and safety of the oral fetal globin inducer, HQK-1001	Reid et al., 2014 [63]
Valproate	Activation of p38 MAPK pathway, HDAC inhibition	Rönndahl et al., 2006 [64]
Thalidomide derivatives	Activation of p38 MAPK pathway	Aerbajinai et al., 2007 [65] Moutouh-De Parseval et al., 2008 [66]
Trichostatin A	HDAC inhibition	Marianna et al., 2001 [67]
Oridonin	Activation of p38 MAPK signaling	Guo L et al., 2020 [68]
Rapamycin (sirolimus)	mTOR inhibitor	Fibach et al., 2006 [69] Zuccato et al., 2022 [70]
Everolimus	mTOR inhibitor	Zuccato et al., 2007 [71]
Mithramycin	Inhibition of Sp1/DNA interactions	Fibach et al., 2003 [72]
Resveratrol	Activation of p38 MAPK signaling	Fibach et al., 2012 [73]
Angelicin	Induction of γ-globin expression via NRF2/ARE stress response pathway	Lampronti et al., 2003 [74]
Cinchonidine, Quinidine and Cinchonine	*Cinchona* alkaloids are potent inducers of the expression of γ-globin genes in erythroid cells	Iftikhar et al., 2019 [75] Zuccato et al., 2021 [76]
Tranylcypromine	Lysine-specific demethylase 1 (LSD1) inhibition	Shi et al., 2013 [77]
Vorinostat	HDAC inhibition reduces the expression of α-globin, induces γ-globin expression	Mettananda et al., 2019 [78]
Vasicinol and Vasicine	Induction of γ-globin genes in a pre-clinical study of HbF inducers isolated from *Adhatoda vasica*	Iftikhar et al., 2022 [79]
Hydroxyurea	Clinical trials NCT00001958 and NCT03183375	Yasara et al., 2022 [80]
HQK-1001	Clinical trial NCT00790127	Reid et al. [63]
Thalidomide	Clinical trial NCT05132270	Ansari et al. [81]
Sirolimus	Clinical trials NCT03877809 and NCT04247750	Gamberini et al., 2021 [82]

**Table 2:** Examples of non-coding RNAs regulating γ-globin gene expression.

Non-Coding RNA	Target mRNA	Comments	Reference
miR-34a	*STAT3*	Stable expression mediates fetal hemoglobin induction	Ward et al., 2016 [138]
miR-210-3p	*BCL11A*	High expression associated with high γ-globin mRNA content; down-regulated BCL11A in erythroid precursor cells induced with mithramycin	Gasparello et al., 2017 [143]
miR-92a-3p	*BCL11A*	Upregulates γ-globin expression and inhibits oxidative stress and apoptosis in erythroid precursor cells	Li H et al., 2022 [144]
miR-30a	*BCL11A*	Regulation of γ-globin gene expression through targeting BCL11A	Gholampour et al., 2020 [145]
miR-2355-5p	*KLF6*	Upregulates γ-globin gene expression	Cheng et al., 2021 [146]
miR-144	*NRF2*	Regulates oxidative stress tolerance of thalassemic erythroid cells	Srinoun et al., 2019 [147]
miR-326	*EKLF*	Regulates HbF synthesis by targeting EKLF in human erythroid cells	Li et al., 2018 [148]
miR-155	*BACH1*	Enhances phagocytic activity of β-thalassemia/HbE monocytes	Srinoun et al., 2017 [149]
miR-26b	*MYB* 3′UTR	Upregulates HbF expression in erythroleukemic K-562 cell line.	Pule et al., 2016 [150]
miR-29b-3p	*Sp1*	Mediates epigenetic mechanisms of HBG gene activation	Starlard-Davenport A 2019 [151]
miR-486-3p	*BCL11A*	Regulation of γ-globin gene expression through direct modulation of BCL11A	Lulli et al., 2013 [152]
miR-15a and miR-16-1	*MYB*	Elevates fetal hemoglobin expression	Sankaran et al., 2011 [153]
miR-190b-5p	*BCL11A*	Regulates BCL11A expression	Chen et al., 2023 [154]
circ-0008102	tbd (*)	May serve as a novel clinical biomarker in beta-thalassemia	Chen et al., 2024 [155]
lncRNA H19	tbd (*)	Leads to upregulation of γ-globin gene expression	Xie et al., 2024 [156]
lncRNA HBBP1	*ELK1*	Leads to upregulation of γ-globin gene expression	A et al., 2021 [157]
lncRNA HMI	tbd(*)	Regulates human fetal hemoglobin expression	Morrison et al., 2018 [158]

(*) tbd = To be determined.

**Table 3:** Examples of pharmacogenomics gene–drug association: drugs employed or proposed for SCA therapy.

Gene/ Biomarker	Drug	Drug RxNorm ID	Medical Intervention (Established or Proposed)	FDA Labeling Sections	CPIC Guideline Classification of Recommendation	Reference from the Literature
*CYP2D6*	Codeine	2670	Pain management	Boxed Warning, Warnings and Precautions, Use in Specific Populations, Patient Counseling Information	https://cpicpgx.org/guidelines/guideline-for-codeine-and-cyp2d6/ (accessed on 25 March 2024)	Gammal et al. (2016) [194]
*CYP2C9*	Ibuprofen	5640	Anti-inflammatory analgesics	n.a. (*)	https://cpicpgx.org/guidelines/cpic-guideline-for-nsaids-based-on-cyp2c9-genotype/ (accessed on 25 March 2024)	Cho et al. (2016) [204]
*CYP3A4*	Rosuvastatin	301542	Reduction in serum cholesterol and cardiovascular complications	Clinical Pharmacology	https://cpicpgx.org/guidelines/cpic-guideline-for-statins/ (accessed on 25 March 2024)	Adam et al. (2013) [205]
*OTC*, *POLG*	Valproic acid	11118	Fetal hemoglobin induction	Boxed Warning, Contraindications, Warnings and Precautions; Testing required	n.a. (*)	Selby et al. (1997) [206]
CYP3A5	Sirolimus	35302	Fetal hemoglobin induction	n.a. (*)	Provisional (accessed on 25 March 2024).	Zuccato et al. (2022) [70]
*ACTP2*, *ERCC1*, *TPMT*	Cisplatin	2555	Fetal hemoglobin induction, Bone marrow transplantation	Adverse Reactions	Provisional (accessed on 25 March 2024).	Lampronti et al. (2006) [207]
*CYP2D6*	Quinidine	9068	Fetal hemoglobin induction	Warnings and Precautions, Clinical Pharmacology	Provisional (accessed on 25 March 2024).	Zuccato et al. (2021) [76]
*CYP2D6*	Quinine	9071	Fetal hemoglobin induction	Drug Interactions, Warnings and Precautions	Provisional (accessed on 25 March 2024).	Zuccato et al. (2021) [76]

(*) n.a. = not available.

## Data Availability

Not applicable.
